# A unique case of tachycardia‐mediated cardiomyopathy in a patient misusing anabolic steroids

**DOI:** 10.1002/ccr3.5976

**Published:** 2022-06-13

**Authors:** Govinda Adhikari, Ashiya Khan, Nasheed Shams, Mustafa H. Hassan, Arvind Kunadi

**Affiliations:** ^1^ Department of Internal Medicine McLaren‐Flint/MSU Flint Michigan USA; ^2^ Department of Cardiology McLaren‐Flint/MSU Flint Michigan USA

**Keywords:** anabolic steroids, cardiomyopathy, case report

## Abstract

A 54‐year‐old male patient with history of anabolic androgenic steroid (AAS) misuse presented to the emergency department with new‐onset atrial fibrillation and severely reduced ejection fraction. Cardiac catheterization revealed normal coronaries. He underwent cryo‐balloon ablation with subsequent conversion to sinus rhythm. After appropriate guideline‐directed medical management, ejection fraction improved on follow‐up.

## Graphical abstract

Cardiomyopathy can be caused by various etiologies including tachycardia and anabolic steroids. A careful history and follow‐up of patients is vital to identify the correct etiology of cardiomyopathy in patients who are misusing anabolic steroids.

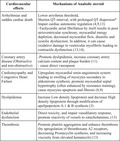

**Learning objectives**

- To understand the cardiovascular effects of Anabolic androgenic steroids (AAS) and its mechanism,
- To understand the growing prevalence of AAS misuse and diagnostic challenge it can impose on the clinicians due to concomitant misuse of other drugs
- To differentiate tachycardia‐mediated cardiomyopathy from AAS‐related and other cardiomyopathies

## 1 INTRODUCTION

Anabolic androgenic steroids (AAS), simply referred to as anabolic steroids, are synthetic forms of the male sex hormone testosterone. They are the most widely misused class of appearance and performance enhancing drugs.^1^ Male non‐athlete weightlifters in 20s or 30s are the major groups of people who misuse steroids in United States.^1^, ^2^ Illicit use of AAS started to grow in late 1970s and early 1980s.^3^, ^4^ There is limited evidence to estimate true prevalence of AAS users in the United States.^1^, ^5^ Projecting the national household survey data, lifetime prevalence of AAS abuse in 2013 was estimated to be around 2.9–4 million.^5^

AAS have been associated with number of cardiovascular diseases including coronary artery disease (both atherosclerotic and non‐atherosclerotic), fatal arrythmias, cardiomyopathy, hypertension, and stroke.^4^, ^6^, ^7^ The exact mechanism of how AAS cause cardiomyopathy and other cardiovascular diseases is still unknown. They are thought to upregulate myocardial renin‐angiotensin system, with resultant aldosterone synthesis leading to myocardial growth and swelling.^8^ AAS are also shown in experimental studies to impair redox sensing mechanism, lower arrythmia threshold, increase low density lipoprotein synthesis and promote apoptosis of myocytes and endothelial cells and fibrosis.^4^, ^9^ We report a case of AAS misuse presenting with atrial fibrillation with rapid ventricular response, and cardiomyopathy in a young patient.

## 2 CASE PRESENTATION

**FIGURE 1:**
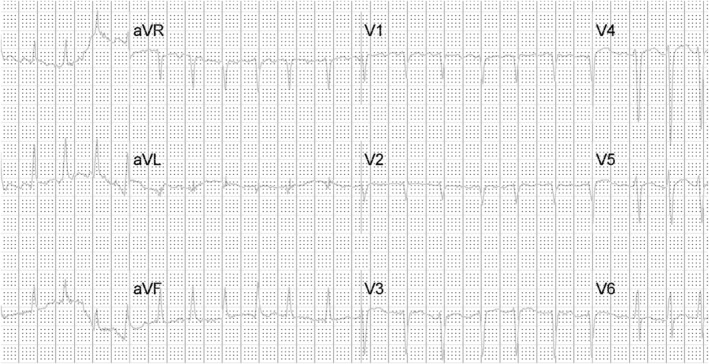
EKG on arrival to the ED showing Atrial fibrillation with RVR, QRS duration 94 ms, Qtc 441 ms 755 × 387 mm (38 × 38 DPI)

A 54‐year‐old Caucasian male with a past medical history of bronchial asthma and polycythemia presented to our emergency department with progressive exertional shortness of breath for 3 weeks, swelling of extremities, and cough with hemoptysis (2–3 episodes) for 1 day. He reported phlebotomies done in the past at the hematologist's office. Family history was significant for atrial fibrillation in the father, but no known cardiomyopathy. He reported of drinking 2–3 beers on weekends and denied smoking or recreational drug use. Review of systems was negative for recent fever, flu‐like illness, recent travel, but positive for generalized fatigue, weakness, lack of energy, 20 pounds weight gain, snoring, and non‐refreshing sleep. On physical examination, he had bibasilar crackles and decreased breath sounds with bilateral pedal pitting edema. On arrival to the emergency department, he was afebrile with blood pressure of 125/83, heart rate:150/min, and saturating 96% in room air. Electrocardiogram showed atrial fibrillation with the rapid ventricular response (ventricular rate 160) and normal QRS duration, intervals, and axis (Figure 1). Chest X‐ray showed bilateral congestion and pleural effusion. CT chest with contrast demonstrated pulmonary edema and pleural effusion, with no evidence of pulmonary embolism.

**FIGURE 2:**
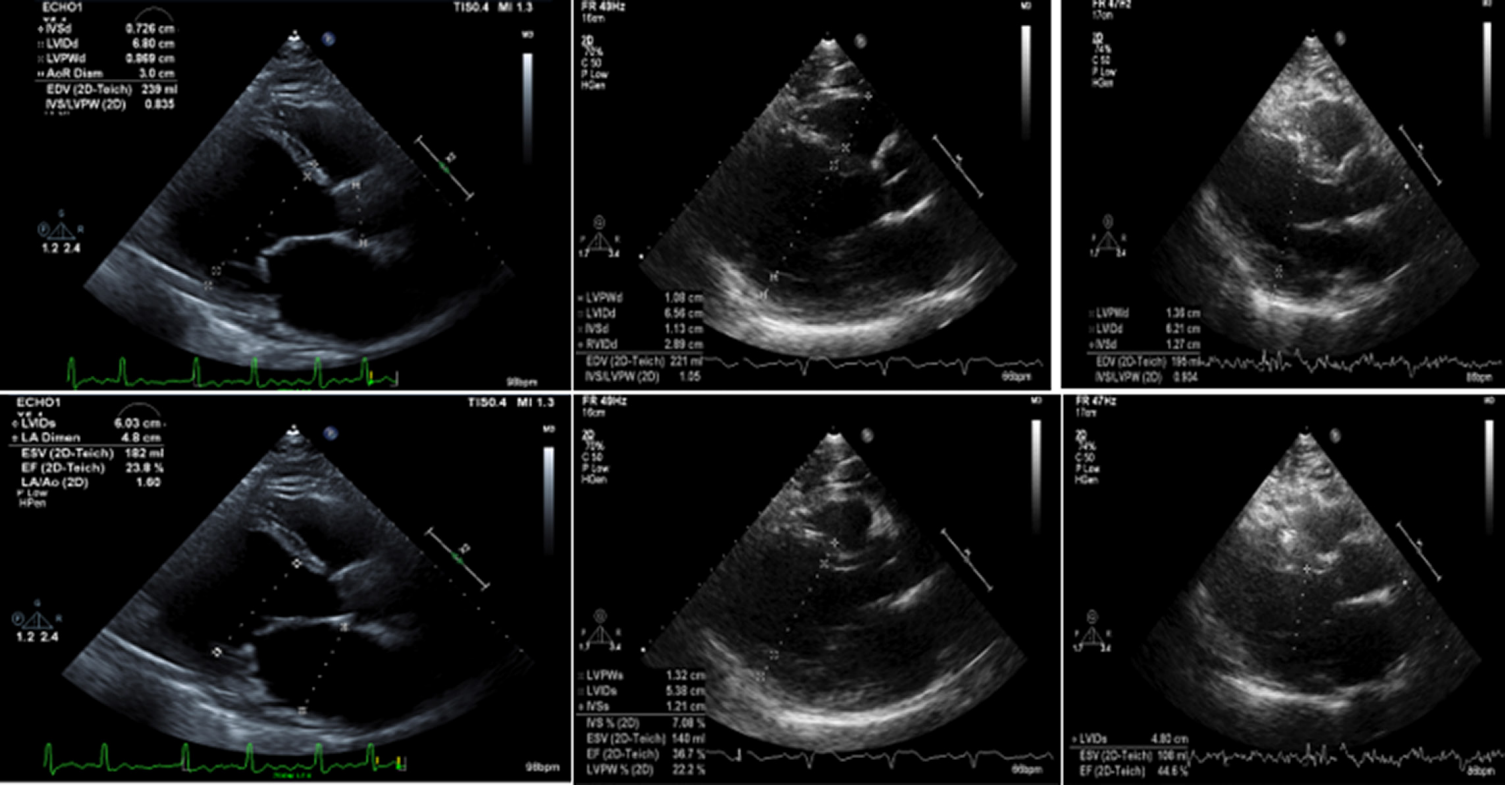
Echocardiogram showing severely reduced LVEF and dilated Left ventricle and atria on the left panel (on admission) with subsequent improvement of LVEF to 44% (at 12 months) on the right panel. The middle panel of echo was obtained at 3 months showing improvement in LVEF and heart dimensions. The upper panels of images were obtained at end‐diastole and lower panels at end‐systole. 563 × 304 mm (38 × 38 DPI)

**FIGURE 3:**
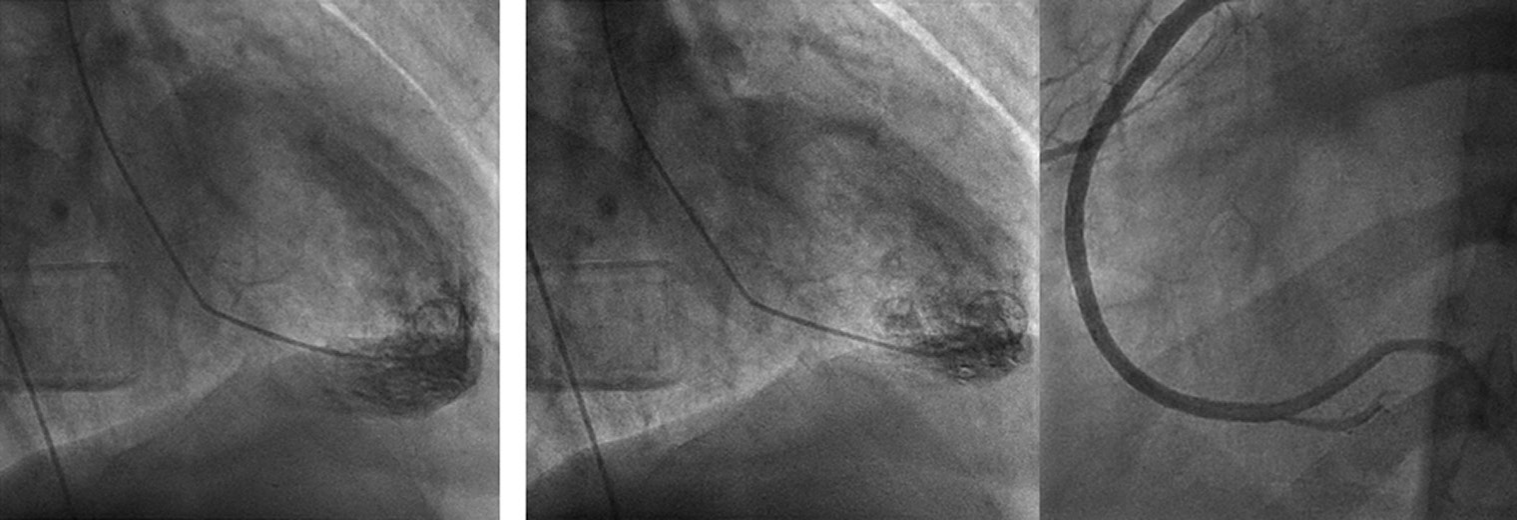
Left Ventriculogram Diastole, Systole, and Left anterior oblique view (from left to right) showing LVEF of 13% 632 × 224 mm (38 × 38 DPI)

He was given a dose of furosemide 40 mg intravenous, started on diltiazem and heparin drip. An echocardiogram was obtained, which showed: Increased left ventricular cavity size with normal thickness and severely reduced systolic function, global hypokinesis, and dilated atria. Left ventricular ejection fraction was 15%–20% (Figure 2). Diltiazem was switched to amiodarone drip later as he developed hypotension. Heart catheterization showed normal coronaries (Figure 3), and an initial diagnosis of non‐ischemic cardiomyopathy with congestive heart failure was made. Further workup showed normal thyroid hormone, anti‐nuclear antibody, iron studies, negative JAK‐2, and elevated erythropoietin (Table 1). Lisinopril, spironolactone, metoprolol was started, and diuresis was continued. He had persistent symptomatic atrial fibrillation and underwent cryo‐balloon isolation of all four pulmonary veins. After the voltage map documented isolation of all four pulmonary veins, patient was still in atrial fibrillation and was cardioverted to normal sinus rhythm using a one‐time 360 joule shock. Subsequently, programmed stimulation during isoproterenol infusion failed to show any evidence of atrial fibrillation or flutter. He tolerated the procedure with no immediate complications. Medical records obtained from his hematologist's office mentioned that he was using intramuscular injections of testosterone cypionate for more than 18 years for bodybuilding. Testosterone levels 3 years ago was 1761 ng/dl. On further questioning, he admitted using testosterone shots and tamoxifen and raloxifene for many years, the last use being 1 month ago. Laboratories showed total testosterone 2060 ng/dl (250–1100 ng/dl); free testosterone 810.5 pg/ml (46–224 pg/ml). He was discharged on lisinopril 2.5 mg daily, spironolactone 25 mg daily, metoprolol 12.5 mg twice a day, apixaban 5 mg twice a day post‐ablation (to prevent the risk of stroke and thromboembolism from left atrial manipulation during the procedure, his CHADSVASc score was 1), amiodarone 200 mg daily. He was also referred to the cardiac rehab program. In addition, he was advised to stop AAS use.

**TABLE 1:** Important laboratory findings on admission

Hemoglobin 18.2 g/dl	BUN 19 mg/dl	TIBC 266 μg/dl	Vitamin B 121193 pg/ml
Hematocrit 52.7%	Creatinine 1.22 mg/dl	Iron saturation 23.68%	Coombs's test, and Anti‐nuclear antibody negative
BNPEP 384 pg/ml	TSH 1.19 mIU/L	IgA 146 mg/dl (60–350 mg/dl)	Cytoplasmic ANCA negative
Troponin 0.03 ng/ml	Magnesium 2 mg/dl	IgG 730 mg/dl (700–1600 mg/dl)	Perinuclear ANCA negative
Total bilirubin 1.5 mg/dl	Potassium 3.6 mM/L	IgM 62.2 mg/dl (40–280 mg/dl)	Total testosterone 2060 ng/dl (250–1100 ng/dl)
AST 25 U/L	Phosphorus 3.3 mg/dl	C3 complement 113 mg/dl (80–207 mg/dl)	Free Testosterone 810.5 pg/ml (46–224 pg/ml)
ALT 30 U/L	Ferritin 101 ng/ml	C4 complement 23.9 mg/dl (10–53 mg/dl)	Erythropoietin 30.27 mIU/ml (2–30 mIU/ml)
ALP 25 U/L	Iron 61 μg/dl	Serum immunofixation: No monoclonal paraprotein	JAK 2 PCR not detected

Follow‐up course:

- 1 week: The patient reported improvement in symptoms. He was in sinus rhythm and admitted stopping testosterone supplements. Amiodarone was discontinued. Sleep study: showed sleep stages: stage R (Rapid Eye Movement): 26.7%, stage N1 (Non‐REM1): 7.6%, stage N2 (Non‐REM2): 65.7%, stage N3 (Non‐REM3): 0.0% total apnea–hypopnea index of 2.6 per hour, total respiratory efforts‐related arousals 2, and total sleep periodic limb movement index of 67.7 per hour.
- 2–12 months: Apixaban was discontinued at 2 months post‐ablation, and he was advised to continue aspirin. Repeat echocardiogram revealed mildly reduced left ventricular function with ejection fraction improved to 36% at 3 months, 41% after 7 months, and 44% at 12 months; normal left ventricular dimension with mild concentric hypertrophy, and borderline left atrial enlargement (Table 2, Figure 2). The patient had stopped taking AAS.

**TABLE 2:** 2‐D Echocardiographic parameters on admission and on follow‐up

Parameters (Normal value)	On admission	3 months	12 months
LV end diastolic volume (67–155 ml)	269 ml	236 ml	214 ml
LV end systolic volume (22–58 ml)	245 ml	145 ml	114 ml
LV internal diameter end‐diastole (4.2–5.9 cm)	9.2 cm	6.76 cm	6.21 cm
LV internal diameter end‐systole (2.1–4.0 cm)	8.7 cm	5.46 cm	4.8 cm
Left ventricular ejection fraction	15%–20%	36%	44%

## 3 DICSUSSION

Testosterone has 1:1 anabolic (muscle‐building): androgenic (masculinizing) properties, while its synthetic derivatives vary vastly in this ratio. However, the more anabolic the steroid is, the more strongly it is associated with organ dysfunction. Free testosterone can diffuse through the lipid membrane into target cells. Once inside a cell, it exerts its effects via the genomic pathway by binding to cytoplasmic receptors and gene transcription and non‐genomic pathways through kinases and intracellular calcium.^10^ Studies have shown a modest association of low endogenous testosterone with increased cardiovascular risk and mortality.^10^ On the contrary, the use of high amounts of exogenous AAS has also been linked to adverse cardiovascular outcomes, as discussed in Table 1.

Cardiomyopathy is a myocardial disorder in which the heart muscle is structurally and functionally abnormal, in the absence of coronary artery disease, hypertension, valvular disease, and congenital heart disease sufficient to cause the observed myocardial abnormality.^11^ AAS can lead to various heart structure and function alterations, namely, myocardial hypertrophy, increased heart chamber size, impairment of contractile, and relaxation function.^12^ Various case reports have reported hypertrophic, dilated, and takotsubo cardiomyopathies with anabolic steroids use. The presence of increased left ventricular cavity size with normal thickness, and severely impaired systolic function in the absence of valvular disease, and no history of hypertension and normal coronaries on heart catheterization led to initial diagnosis of non‐ischemic cardiomyopathy in our case.

The patient had no history of intravenous drug abuse. Alcohol intake of >80 g per day for at least 5 years is known to cause alcoholic cardiomyopathy. However, our patient reported drinking alcohol occasionally. Viral etiology was unlikely because of the absence of fever, no recent flu‐like illness, normal leukocyte count. Influenza A and B PCR was negative. He was not on any medications known to cause cardiomyopathy. He had normal thyroid function, iron studies, and electrolytes. Autoimmune, vasculitis, and paraprotein screen were negative. The sleep study done a month after discharge from the hospital showed a mild tendency towards obstructive respiratory events with an overall respiratory disturbance index of 2.9 per hour suggesting a low probability of sleep apnea. He instead had periodic limb movements during the test, which was the likely cause of his non‐refreshing sleep. In addition, our patient presented in atrial fibrillation and secondary polycythemia (supported by elevated erythropoietin level, with negative JAK2 and no evidence of other causes of secondary polycythemia). The dilemma appeared whether the cardiomyopathy was tachycardia‐mediated or from the direct toxic effect of AAS. AAS can cause cardiomyopathy by the mechanism shown in Table 3. Although our patient never had prior documented atrial fibrillation and had an existing potential cause of cardiomyopathy, that is, AAS, the resolution of atrial fibrillation and subsequent recovery of left ventricular function after catheter ablation and conversion to sinus rhythm points towards the diagnosis of tachycardia‐mediated cardiomyopathy in our case. However, the possible contribution of AAS itself being a potential trigger for atrial fibrillation and cardiomyopathy cannot be completely excluded. In addition, the sustained left ventricular dysfunction at 12 months following pulmonary vein isolation is likely to imply incomplete recovery due to myocardial injury possibly from AAS use and/or tachycardia.

**TABLE 3:** Cardiovascular effects and proposed mechanisms of action of anabolic steroid^3^, ^4^, ^8^, ^9^, ^11^, ^13^

Cardiovascular effects	Mechanisms of anabolic steroid
Arrhythmias and sudden cardiac death	Lower arrythmia threshold, Shorten QT‐interval, with prolonged QT dispersion^a^ Impair cardiac autonomic regulation ^4^, ^9^, ^13^ Tachycardic atrial fibrillation by itself results in poor atrioventricular synchrony, myocardial energy depletion, decreased myocardial flow, diastolic and systolic dysfunction. In addition, it can cause oxidative damage to ventricular myofibrils leading to contractile dysfunction^13^, ^14^
Coronary artery disease (Obstructive and non‐obstructive)	Promote dyslipidemia, increase coronary artery calcium content and plaque burden^11^ cause direct vasospasm
Cardiomyopathy and Congestive Heart Failure	Upregulate myocardial renin‐angiotensin system leading to swelling of myocytes secondary to aldosterone synthesis, promote myocardial septal hypertrophy (often enhanced by resistance training), cause myocytes apoptosis and fibrosis^8^, ^9^
Dyslipidemia	Increase Low density lipoprotein and decrease High density lipoprotein through modification of apolipoprotein A‐1 & B synthesis^3^
Endothelial dysfunction	Direct toxicity, and impair vasodilatory response, promote reactivity of vessels to catecholamines^13^
Thrombosis	Promote platelet aggregation and enhance thrombosis (by upregulation of thromboxane A2 receptors, decreasing Prostacyclin synthesis, and increasing viscosity from elevated hematocrit)^13^
Blood pressure elevation	Increase production of deoxycorticosterone levels through inhibition of 11 beta hydroxylase activity, increase synthesis of renin by acting at genomic level, and impair vascular function^4^, ^13^

^a^ QT dispersion: the difference between longest and shortest corrected QT‐interval in a 12 lead EKG.

Tachycardia‐mediated cardiomyopathy is known to resolve with treatment of underlying tachycardia. However, some patients with underlying fibrosis are less likely to recover fully and are predisposed to an increased risk of sudden cardiac death.^13^ Cardiac MRI may play an essential role in identifying such high‐risk patients. The pathophysiology of the cardiomyopathy has not been fully elucidated yet. Tachycardiac atrial fibrillation can cause impaired myocardial blood flow and depletion of energy stores. Chronic tachycardia causes myocyte loss, myocyte elongation, depletion of t‐tubules at the microscopic level resulting in contractile dysfunction.^13^, ^14^ The possible role of oxidative damage to myofibrils and genetic factors, including angiotensin‐converting enzyme polymorphism, has also been hypothesized.^13^ Testosterone is known to cause polycythemia by direct bone marrow stimulation and alteration in erythropoietin set point.^15^ Atrial fibrillation might have resulted from cardiomyopathy and/or the effects of AAS.

The diagnosis of cardiomyopathy is challenging to make. There are several potential limitations to our case report. It is important to understand that there is also a possibility of having undiagnosed idiopathic dilated cardiomyopathy before the episode of tachycardia‐induced acute heart failure that recovered with appropriate guideline‐directed medical therapy. The echocardiogram obtained during the episode of atrial fibrillation and the slanted cross‐section of the M mode at 12 months limit the reliable evaluation of left ventricular function. The initial worsening of hypotension in our case was probably due to negative chronotropic action of diltiazem which was inappropriately used in the setting of acute heart failure in the emergency department.

## AUTHOR CONTRIBUTIONS

All authors were involved in the conception and design, critical revision, final approval, and agreed to be accountable for all aspects of the work. Authors 1,2, and 3 were involved in initial drafting of the manuscript.

## CONFLICT OF INTEREST

None.

## CONSENT

Written informed consent was obtained from the patient to publish this report in accordance with the journal's patient consent policy.

## Supporting information


Video S1

## Data Availability

Data available on request from the authors.
